# Occurrence of Antibiotic-Resistant Bacteria in Therapy Pools and Surrounding Surfaces

**DOI:** 10.3390/ijerph15122666

**Published:** 2018-11-27

**Authors:** Daniela E. Koeck, Stefanie Huber, Nadera Hanifi, Manfred Köster, Martina B. Schierling, Christiane Höller

**Affiliations:** 1 Bavarian Health and Food Safety Authority, Veterinärstraße 2, 85764 Oberschleißheim, Germany; stefanie.huber@lgl.bayern.de (S.H.); Nadera.hanifi@lgl.bayern.de (N.H.); Martina.Schierling@lgl.bayern.de (M.B.S.); christiane.hoeller@lgl.bayern.de (C.H.); 2 Bavarian Health and Food Safety Authority, 91058 Erlangen, Germany; Manfred.koester@lgl.bayern.de

**Keywords:** antibiotic resistance, hydrotherapy, pool, water, treatment, transfer

## Abstract

The number of patients colonized with antibiotic-resistant bacteria is increasing in health care facilities. Because transmission of antibiotic-resistant bacteria is feared, there exist reports that the affected patients are frequently excluded from hydrotherapy, which is a non-invasive and beneficial treatment used for patients with different diseases. Data from the literature suggest that deficient water disinfection measures exist, which are not always sufficient to kill all released bacteria. If the pool water is not disinfected properly, it may also infect the bathers. Immunocompromised patients are particularly susceptible to be infected with (antibiotic-resistant) bacteria. In order to determine the distribution of antibiotic-resistant bacteria in the pool water treatment system and the pool environment and to estimate the associated transmission risk we analyzed samples from eleven health care facilities. Antibiotic-resistant bacteria were found in the water and surface samples collected. One hundred and two antibiotic-resistant isolates from water samples and 307 isolates from surrounding surfaces were obtained, respectively. The majority of the isolates belonged to non-fermenting Gram-negative rods, like *Pseudomonas* spp. Some isolates were resistant to a wide range of the tested antibiotics. The results indicate a relation between the number of isolates in water samples and the number of patients using the pools in combination with deficiencies in water treatment. In the pool environment the highest number of isolates was obtained from barefoot areas and floor cleaning equipment.

## 1. Introduction

Emerging and increasing antibiotic microbial resistance (AMR) represents one major threat to human health in Europe and worldwide. Resistance to antibiotics is widely distributed among Gram-positive and Gram-negative bacteria that may cause serious infections in humans, and AMR is increasing in the EU, especially among Gram-negative bacteria. The major drivers behind the occurrence and spread of AMR are the use of antimicrobial agents and the transmission of antibiotic-resistant microorganisms between humans; between animals; and between humans, animals, and the environment [1].

Bacteria that are resistant to three or more classes of antibiotics are called multidrug-resistant. Infections with these bacteria are associated with increased morbidity, mortality, length of hospitalization, and financial costs [2]. For a long time, methicillin-resistant *Staphylococcus aureus* (MRSA) have been the main point of concern in public health, but during the last couple of years extended-spectrum-ß-lactamase (ESBL)–producing bacteria have become a much more severe problem. While MRSA are predominantly acquired in connection with medical treatments, the picture concerning multidrug-resistant Gram-negative bacteria is much less clear and differs from species to species [3]. The production of ESBL is the most frequent resistance mechanism among Gram-negative bacteria. The corresponding gene sequences of the ESBLs are mostly on mobile DNA elements and can be transmitted horizontally, which contributes to the spread of these resistance genes [4]. Due to the resistance of the microorganisms to β-lactam antibiotics and other classes of antibiotics such as the fluoroquinolones (e.g., ciprofloxacin and levofloxacin), the therapeutic spectrum is strongly restricted in the case of infection with ESBL-producing bacteria [5]. Options for treatment of patients who are infected with multidrug-resistant bacteria are limited to only a few remaining last-line antibiotics, such as carbapenems (e.g., imipenem, ertapenem, meropenem). However, the increasing carbapenem-resistance limits options for the treatment of infected patients [6,7]. For the KRINKO (German Commission on Hospital Hygiene and Infection Prevention) definition of multidrug-resistant Gram-negative rods (MRGN), only resistance to antibiotics which are used as primary therapeutics for severe infections (acylaminopenicillins, third and fourth generation cephalosporins, carbapenems, and fluoroquinolones) has been included: 3MRGN (with resistance to three of the four antibiotic groups) and 4MRGN (with resistance to four of the four antibiotic groups). Aminoglycosides, like amikacin, gentamicin, and tobramycin, were not included in the KRINKO classification of multidrug-resistant Gram-negative rods, as they are generally not used as monotherapeutics [8].

Hydrotherapy is a non-invasive and beneficial treatment for many patients, like patients with chronic diseases, disabilities, or trauma. Very often those patients have a long history of medical treatments, including antibiotic treatments. Thus, the probability that they have a disturbed microflora with an increased rate of carriage of AMR and also a reduced colonization resistance against AMR is relatively high [9]. Although pool water is usually disinfected, infections are known to occur either due to deficiencies in water treatment or due to colonization of swimming pool equipment [10,11,12,13]. Therapies performed in a swimming pool cause a large release of bacteria. Bathers transfer approximately 10^5^–10^6^ CFU per person in 15 min to the surrounding water body [14]. Bacteria should be inactivated by disinfectants in the pool water; however, this is not always the case, because they can be attached to particles or be protected by an EPS (extrapolymeric substance) and thus not be exposed to the disinfectant. Bacteria can form biofilms on pool surfaces, especially in areas of the pool where the concentration of disinfectant is low [15,16,17]. Additionally, they can be attached to swimming aids, which are made from plastics, often foams, which provide a large surface. They can also be found on tools for cleaning, where they are exposed occasionally but not permanently to disinfectants. *Pseudomonas aeruginosa*, a species very often involved in biofilm formation, has been commonly isolated from the pool environment [18,19].

Immunocompromised patients are particularly susceptible to infections with pathogens (including antibiotic-resistant bacteria) via the mucous membranes and via penetration of bathing water into auditory canals and the nasopharynx. Although some literature exists on antibiotic-resistant bacteria in swimming pools [16,17,20], little is known about the prevalence of AMR. To this day, to our knowledge, no information exists about the public health impact of therapy pools in the dissemination of antibiotic-resistant bacteria. In order to assess the extent of contamination with antibiotic-resistant bacteria, their distribution, and the associated transmission risk in clinical therapy pools, we performed a study on this topic. The main objective of the project was to investigate the occurrence of antibiotic-resistant bacteria in water of therapy pools, in filters, balance tanks, and on surrounding surfaces. Factors contributing to their occurrence will be discussed with regard to details in pool water treatment and disinfection, number of patients entering the pools, and usage of the pools for other purposes. Finally, we derive recommendations for the management of patients colonized with antibiotic-resistant bacteria in hydrotherapy pools.

## 2. Materials and Methods

### 2.1. Sampling

Eleven pre-selected therapy pools located in different hospitals in Bavaria were sampled in accordance with the requirements of DIN EN ISO 19458 [21]. Bottles with capacities of 250 and 1000 mL, prepared with sufficient (100 mg/L) sodium thiosulfate (Na_2_S_2_O_3_, sodium thiosulfate pentahydrate, Merck, Darmstadt, Germany) for dechlorination, were used. Pool water and balance tank water samples were collected from a depth of 30 cm, at a point about 40 cm away from the basin edge, filtrate was taken from a sampling tap, and filter backwash water was taken directly before the drain. The samples were transferred to the laboratory within 1–2 h from collection, using appropriate insulated coolers, and they were processed immediately after arrival at the laboratory. In addition to the water samples, samples from the surrounding surfaces (especially sanitary areas and pool equipment) were taken with sterile, wet swabs, moistened with 0.9% NaCl (w/v). All sampling sites are summarized in Table 1. The swabs were transported to the laboratory immediately after the collection. In order to isolate microorganisms, the tips of the swabs were cut off and placed in tubes containing 10 mL of sterile CASO-broth (BD BBL™ Trypticase™ Soy Broth, Becton Dickinson GmbH, Heidelberg, Germany). The tubes were vortexed (for 2 min) to remove microbial cells from the swab material and incubated for 24 h +/− 2 h at 37 °C. Four samples of each sampling point (water and surfaces) were collected over the course of one year (one sampling per quarter).

### 2.2. Determination of Water Quality Parameters According to DIN 19643-1

Typical water quality parameters (chemical and microbial) were determined from all water samples according to DIN 19643-1 [21]. The parameter limits and all references for the methods used are listed in Table 2.

For the measurement of chlorates and chlorites by LC-MS, a hypercarb column (100 × 2.1 mm, 5 μm, Thermo Scientific, Waltham, MA, USA) was used. The eluents used were deionate/methanol 95/5 with 1% (w/v) formic acid (A) and methanol with 1% (w/v) formic acid (B). The flow rate was 0.3 mL/min with an injection rate of 10 μL. For subsequent mass spectrometry with the API 5500 device (SCIEX, Darmstadt, Germany), the software package Analyst 1.6.2 (SCIEX, Darmstadt, Germany) was used. For the measurement, a three-point calibration with bracketing was performed. The concentrations of the standards were 10, 50, and 100 μg/L. The samples were diluted according to the calibration line. The internal standard used was ^18^O_3_-chlorate.

To determine the acid capacity (K_S4.3_), the samples were titrated with 0.1 mol/L hydrochloric acid until a pH of 4.3 was reached (DIN 38409-1). The necessary amount of acid was documented and the acid capacity was calculated as follows:

(1) $$\mathrm{Ks}=\frac{a\times 1000\times 0.1\times f}{W}\;$$

where Ks refers to acid capacity, *a* refers to the titrated volume in mL of 0.1 mol/L HCl, *W* refers to the sample volume (100 mL), and *f* refers to the titer of 0.1 m HCl (=1.000).

The oxidation–reduction potential was recorded on site using the continuously operating measuring systems the pools applied to control the necessary addition of chemicals. The measurement was performed with platinum or gold electrodes against a silver/silver chloride reference electrode and the measured voltage was converted to the standard hydrogen electrode.

### 2.3. Determination of the Total Number of CFU (Colony Forming Units) from the Pool Surroundings

Contact plates (ICRplus (Isolators and Clean Rooms) TSA (Tryptic Soy Agar) contact plates with LTHThio neutralizers, Millipore, Darmstadt, Germany) were used to determine the total number of CFU from the surrounding surfaces. All sampling sites are summarized in Table 1. The agar plates were incubated for 24 h +/− 2 h at 37 °C. It is important to ensure that sampling is carried out at representative and similar sites to ensure comparability between the therapy pools. Based on the DGfdB (German Society for Bathing) guideline 94.04 [22], it can be determined whether or not the surface cleaning and disinfection was performed adequately.

### 2.4. Isolation of Antibiotic-Resistant Bacteria

The abovementioned water samples (1000 mL or 100 mL, respectively; Table 1) were filtrated through sterile 0.45 µm membrane filters (Millipore, Darmstadt, Germany). Filters were subsequently placed on MacConkey agar (Oxoid, Wesel, Germany) supplemented with cefotaxime (1 mg/L), Brilliance carbapenem-resistant *Enterobacteriaceae* (CRE) Agar (Oxoid), ChromID™ VRE Agar (bioMérieux, Nurtingen, Germany), and ChromID_TM_ MRSA SMART Agar (bioMérieux, Nurtingen, Germany), and incubated for 24 h +/− 2 h at 37 °C under aerobic conditions. Next, 200 µL from the liquid enrichments of the culture swabs were plated on the same selective media and incubated for 24 h +/− 2 h at 37 °C. One colony of each phenotype was picked from the selective plates, subcultured on 10% defibrinated sheep blood agar (Oxoid), and incubated at 37 °C for 24 h. Identification to the species or genus level was performed using BD Phoenix™ 100 (Becton Dickinson Diagnostic systems, Heidelberg, Germany) and MALDI-TOF-MS Microflex LT (Bruker, Bremen, Germany). The MALDI Biotyper Real Time Classification System (Bruker) and the BD EpiCenter^TM^ Software (Becton Dickinson, Heidelberg, Germany) were used for the identification and taxonomical classification of bacteria.

### 2.5. Antimicrobial Susceptibility Testing

Antimicrobial susceptibility testing was performed by Phoenix™ Panels NMIC 448794 (Becton Dickinson Diagnostic systems, Heidelberg, Germany) for Gram-negative bacteria, with 21 antimicrobial substances (ampicillin, piperacillin, piperacillin-tazobactam, cefuroxime, cefotaxime, ceftazidime, cefepime, imipenem, meropenem, aztreonam, gentamicin, tobramycin, amikacin, ciprofloxacin, levofloxacin, trimethoprim-sulfamethoxazole, amoxicillin-clavulanic acid, ertapenem, fosfomycin+G6P, tigecycline) and Panel NMIC 448796 for Gram-positive bacteria, with 23 antimicrobial substances (penicillin G, ampicillin, oxacillin, cefoxitin, imipenem, clindamycin, erythromycin, vancomycin, teicoplanin, linezolid, fusidic acid, rifampicin, nitrofurantoin, gentamycin, tobramycin, ciprofloxacin, moxifloxacin, tetracycline, trimethoprim-sulfamethoxazole, daptomycin, fosfomycin, gentamicin-syn, tigecycline) according to the manufacturer’s guidelines (Becton Dickinson Diagnostic Systems, Heidelberg, Germany). Results of all antimicrobials tested were interpreted according to the European Committee on Antimicrobial Susceptibility Testing (EUCAST) breakpoints (http://www.eucast.org/clinical_breakpoints). The calculation of MIC50 (representing the minimal inhibitory concentration (MIC) of 50% of the isolates) and MIC90 (representing the MIC of 90% of the isolates) were calculated using the obtained values from Phoenix™.

### 2.6. Questionnaire

A standardized questionnaire was developed and given to the facilities operating the therapy pools to document the technical details of the pool, their water treatment, cleaning procedures, and frequency and duration of pool usage (Supplementary Materials S1).

### 2.7. Statistical Analysis

Pearson’s χ^2^ test was used to test whether observed differences in contamination of water samples and surface samples between the sampling locations were statistically significant. An interactive calculation tool for chi-square tests (available from http://quantpsy.org) was employed for all statistical analyses and the significance level was set at 95% (*p* ≤ 0.05) for all analyses.

## 3. Results

### 3.1. Water Quality Parameters According to DIN 19643-1

The number of bathers/patients using the pool clearly differed between the sampled facilities (ranging from <50 patients per year to 35,000 patients per year). None of the pools exceeded the maximal bathing load per hour (according to DIN 19643-1 [21]). The chemistry of the pool water samples is shown in Table 3. Location six and eight were only sampled twice and location ten only three times, because the bathing facilities were closed down during the study. The results of the filtrate, balance tank water, and filter backwash water samples are not shown, since there are no standard requirements specified in DIN 19643-1 [21]. The filtrate should only be investigated in case of problems with the pool water. Most of the chemical parameters of the pool water met the requirements. Only the acid capacity was often (59%) too low, which is an indicator for a low puffer capacity and may hinder a proper flocculation. The aluminum concentration was above the limit value in 64% of the sampled basins, which is an indication of a flocculation failure due to too much flocculant or an inadequate flocculation (e.g., due to insufficient mixing). Furthermore, a relationship was observed between the high number of visitors (indicated in grey) in pool number two and eight and a distinctly higher concentration of bound and free chlorine.

The microbiological quality of the investigated pools was considered acceptable/unacceptable according to the German standards DIN 19643 [21]. The limits are valid for pool water and filtrate (not for balance tank water and filter backwash water). All pool water samples examined met the microbiological standards specified in Table 2. We detected three exceedances in the filtrate: >12,600/mL CFU total heterotrophic counts (36 °C) and >200 CFU/100 mL *P. aeruginosa* in one sample and again 108/mL CFU total heterotrophic counts (36 °C) in another sample. Both samples came from facility number ten. *E. coli* was not detected in any of the samples analyzed.

### 3.2. Determination of Total Heterotrophic Counts from the Pool Surroundings

An analysis of the samples revealed that 78% of the total plate count samples (370) showed bacterial growth, i.e., were counted as positive (Table 4). One hundred and seventeen samples (32%) exceeded the action value (800 CFU/100 cm^2^) from the DGfdB guideline 94.04 [22]. However, as our samples were taken when bathers were present, these results should be interpreted with caution, because the guideline values refer to areas after cleaning and/or disinfection in order to determine if the surface cleaning and disinfection has been performed adequately.

### 3.3. Bacterial Isolates

**Figure 1:**
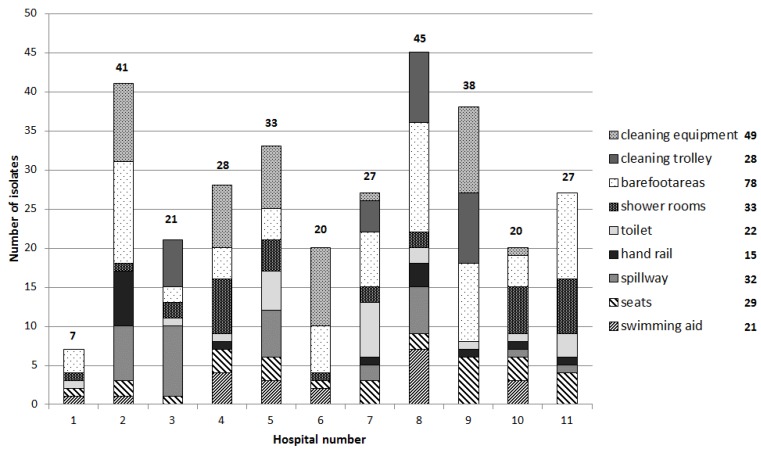
Number of antibiotic-resistant isolates (growth on selective media) from the surrounding surface samples (*n* = 371).

A total of 307 isolates from 23% positive samples (growth on selective media) were obtained from all surface samples (*n* = 371). A significant difference between the surface samples across the sampled pools was observed (*X^2^* = 200.138, *p*-value = 0). Most isolates were obtained from hospital number eight, which was only sampled twice (Figure 1). Hospital number two and five, which have the second and third highest number of pool visitors per year, also yielded a high number of isolates. A relationship was observed between the high number of visitors (Table 3) and the number of isolates. The highest number of isolates was obtained from barefoot areas (78) and floor cleaning equipment (49).

**Figure 2:**
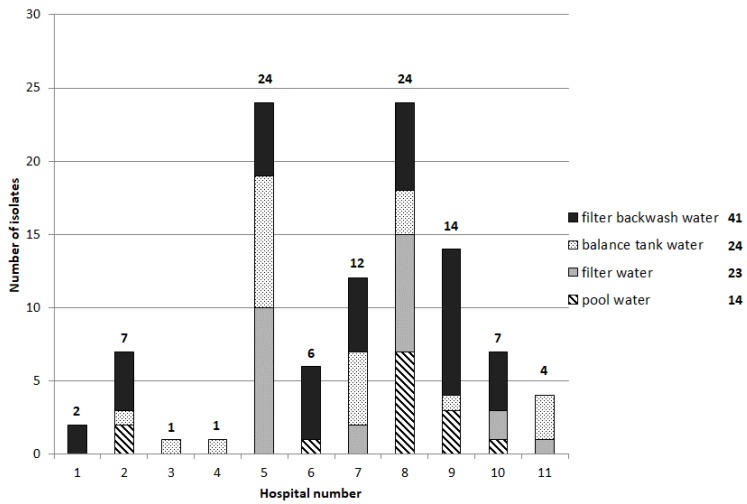
Number of antibiotic-resistant isolates (growth on selective media) from the water samples (*n* = 155).

A total of 102 isolates from 32% positive samples (growth on selective media) were obtained from all water samples (*n* = 155) (Figure 2). A significant difference between the water samples across the sampled pools was observed (*X*^2^ = 52.778, *p*-value = 0.002). The two therapy pools with the highest number of isolates (number five and number eight) also had the highest number of visitors, even if pool number eight was only sampled twice. Most of the positive water samples were from the balance water and filtrate; the pool water itself was contaminated less frequently. Isolates could be obtained directly from the pool water in only pool number two, six, eight, nine, and ten.

**Figure 3:**
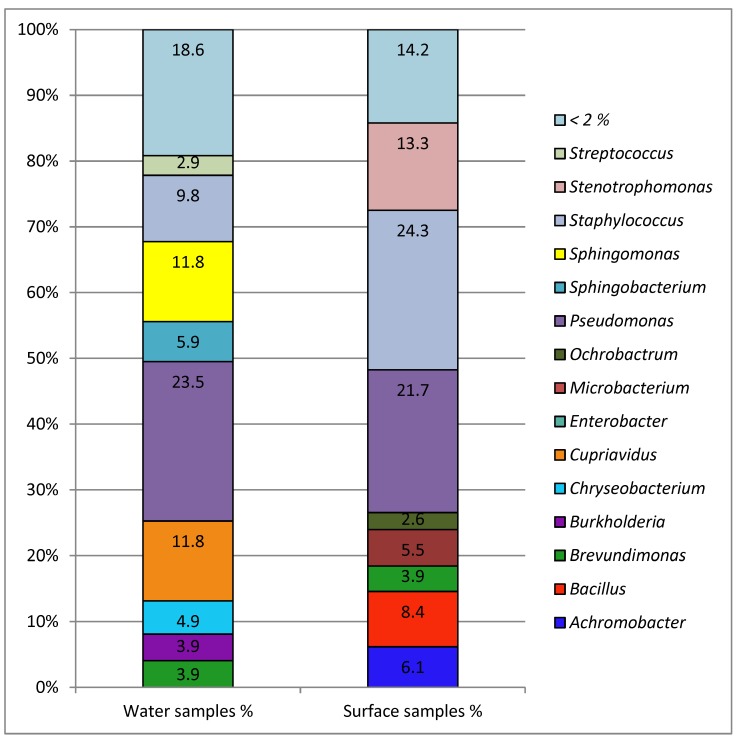
Taxonomic profile up to genus level for all antibiotic-resistant isolates. Left column: percentage of water isolates (*n* = 102); right column: percentage of isolates from surfaces (*n* = 307). Other genera present in <2%. Identification to the species or genus level was performed using BD Phoenix™ and MALDI-TOF-MS.

Not all isolates could be classified up to species level (Figure 3). For genetically closely related species the applied identification methods (BD Phoenix™ and MALDI-TOF-MS) only allowed assignment to the genus level. The majority of the isolates belonged to the taxonomically heterogeneous group of non-fermenting Gram-negative bacteria. Typical genera like Burkholderia spp., *Moraxella* spp., *Pseudomonas* spp., *Stenotrophomonas* spp., and *Sphingomonas* spp. were found. Some of them are typical water borne bacteria, like *Pseudomonas* spp. *E. coli* was not isolated and other coliform bacteria were very rare. *Acinetobacter* spp. were also not isolated. The abundant Gram-positive genera are mostly inhabitants of the natural skin flora (like *Staphylococcus epidermidis)* or environmental bacteria (like *Bacillus subtilis*). There was only one *S. aureus* isolate from a handrail, which was oxacillin sensitive. Some genera appeared in high abundances only in water samples (like *Sphingomonas* and *Sphingobacterium*) and some were mainly found in environmental samples (like *Stenotrophomonas*, *Bacillus*, *Achromobacter, Ochrobactrum*). *Pseudomonas* spp. is one of the most abundant genera in both kinds of samples. Isolates from the pool water (*n* = 14) were mostly *Pseudomonas* spp. (*n* = 3), *Sphingobacterium* spp. (*n* = 4), and *Staphylococcus* spp. (*n* = 4).

### 3.4. Antimicrobial Susceptibility

The isolated Gram-positive genera have no or very rare clinical relevance, therefore the antibiotic resistance patterns (antibiograms) of Gram-positive isolates were not further analyzed. There were abundant Gram-negative genera, like *Pseudomonas, Stenotrophomonas*, and *Sphingomonas*, which can cause infections especially in immunocompromised patients; their antibiograms are shown in Table 5. *Sphingobacterium* and *Achromobacter* are the two other abundant Gram-negative genera. These are rarely involved in human infections; their resistance patterns are shown in Table 6. For these five bacteria genera, the MIC50 (minimal inhibitory concentration (MIC) for 50% of the isolates) and the MIC90 (MIC for 90% of the isolates) were calculated using the obtained values from the Phoenix^TM^ experiments (Table 7 and Table 8).

The antibiograms of other *Pseudomonas* species (e.g., *P. putida*) isolates (*n* = 136), are not shown, as they are seldom associated with infections in humans. The total abundance of all *Pseudomonas aeruginosa* isolates was 5%. Among these isolates five were resistant to ciprofloxacin (MIC50: 0.25 µg/mL; MIC90: 2 µg/mL) and levofloxacin (MIC50: 1 µg/mL; MIC90: 4 µg/mL). Three *P. aeruginosa* isolates were simultaneously resistant to imipenem (MIC50: 2 µg/mL; MIC90: 8 µg/mL), ertapenem (MIC50: 2 µg/mL; MIC90: 2 µg/mL) and all tested fluoroquinolones. But all *P. aeruginosa* isolates were susceptible against piperacillin, ceftazidime and cefepime. The 39 *Stenotrophomonas maltophilia* isolates were resistant to almost all tested antibiotic substances. All isolates were susceptible against trimethoprim-sulfamethoxazole. The susceptibility against polymyxins was not investigated. Among the 14 *Sphingomonas paucimobilis* isolates there existed a high diversity between the resistance patterns. One isolate was susceptible to at least one antibiotic substance of all classes of antibiotics. The majority of the isolates was resistant to all tested carbapenems and sensitive to other classes. But there was also one isolate, which was resistant to 4 of 4 antibiotic groups from the KRINKO classification for MRGN.

The isolates from the genus *Achromobacter* could not be resolved up to species level, as the databases lack appropriate reference spectra. All *Sphingobacterium* spp. isolates belong either to the species *S. spiritivorum* or *S. multivorum*. Some isolates were resistant to a wide range of the tested antibiotics.

## 4. Discussion

The therapy pool with the highest number of isolates obtained directly from the pool water and from the sampled surfaces had not only the highest number of visitors but also seemed to have problems with the water treatment (high bound chlorine levels). The cleaning intervals of the pool areas were the same between the different health care facilities (once per day, according to the evaluation of the questionnaire); hence there is no detectable correlation between the number of isolates and the cleaning interval. These results indicate a correlation of the incidence of antibiotic-resistant bacterial isolates with the number of patients in combination with deficiencies in water treatment. The isolation of potential human pathogens, particularly *P. aeruginosa, S. maltophilia*, and *S. paucimobilis* strains, indicates that these inhabitants of the nosocomial environment may have been released by bathers, with contact to surfaces in the surrounding of the pool and the hospital environment, after entering the pool. *P. aeruginosa* can potentially cause disease in healthy humans, but more often it colonizes immunocompromised patients, like those with cystic fibrosis or cancer [23]. *P. aeruginosa* is intrinsically resistant to the majority of antimicrobial agents due to the low permeability of its outer membrane and the constitutive expression of various efflux pumps. Any additional acquired resistance severely limits the therapeutic options for treating serious infections. The antimicrobial groups that remain active against the susceptible *P. aeruginosa* phenotype include some fluoroquinolones (e.g., ciprofloxacin and levofloxacin), aminoglycosides (e.g., gentamicin, tobramycin, and amikacin), some beta-lactams (piperacillin- tazobactam, ceftazidime, cefepime, imipenem, doripenem, and meropenem), and polymyxins (polymyxin B and colistin). Resistance of *P. aeruginosa* to these agents can be acquired through one or more of several mechanisms, like the acquisition of plasmid-mediated resistance genes coding for various *β*-lactamases and aminoglycoside-modifying enzymes [24,25]. Some strains that have been isolated exhibited resistance to essentially antipseudomonal antibiotics, like fluoroquinolones and carbapenems.

Another problematic nosocomial pathogen is *S. maltophilia,* which is also naturally resistant to many broad-spectrum antibiotics (including all carbapenems)*. S. maltophilia* is the third most common nosocomial pathogen with multi-drug-resistance [26]. *S. maltophilia* is often associated with pulmonary infections, urinary tract infections, bloodstream infections, and colonization of individuals with cystic fibrosis, especially in immunocompromised patients. The treatment of infected patients is very difficult [27,28]. It can be considered positive that all *S. maltophilia* isolates were susceptible against trimethoprim-sulfamethoxazole, as trimethoprim-sulfamethoxazole is still the treatment of choice for suspected or culture-proven *S. maltophilia* infections [29]. If a patient is infected with one of these strains, polymyxins may also be effective treatment options, though not without frequent adverse effects.

*Sphingomonas paucimobilis* has been implicated in various types of clinical infection. Although infections by *S. paucimobilis* are rarely serious and could be effectively treated with antibiotics, *S. paucimobilis* is capable of causing active infections in humans [30,31]. However, 93% of the isolates in this study were resistant to aminoglycosides, and one isolate was resistant to ceftazidime. Another study describes carbapenems, against which all isolates were resistant, as the most effective therapy for infections with *S. paucimobilis* [32]. *Achromobacter* spp. have been identified as opportunistic human pathogens in people with certain immunosuppressive conditions, such as cystic fibrosis, cancer, and kidney failure [33]. Notably, 80% of the isolates originated from barefoot areas or the floor cleaning equipment, which indicates a transmission due to insufficient management of floor cleaning equipment.

Like *Achromobacter* spp., *Sphingobacterium spiritivorum/multivorum* is rarely involved in human infection. Sphingobacterium species are intrinsically resistant to many commonly used antibiotics and can grow in antiseptics and disinfectants [34]. S. multivorum can produce an extended-spectrum *β*-lactamase and a metallo-*β*-lactamase, conferring resistance to third-generation cephalosporins and carbapenems, respectively [35]. Two isolates were both resistant to carbapenems and third-generation cephalosporins.

In addition to these quite important human pathogens, some uncommon antibiotic-resistant Gram-negative bacterial species, like *Chryseobacterium indologenes* and *Ochrobactrum anthropi*, were isolated. The isolation of *Chryseobacterium indologenes* and *Ochrobactrum anthropi* from swimming pool water was described previously by Papadopoulou et al. [17]. In this study, *Ochrobactrum anthropi* could only be isolated from the surface samples in the pool surroundings and not from the water samples. For both species, rare clinical significance and resistance to a wide variety of antimicrobial agents has been reported [36,37].

However, whether such resistant strains can contaminate bathers and cause infection strongly depends on the immune status of the patient. The study has revealed deficiencies in the operation of the pools, although the extent to which immunocompetent patients may be at risk from multidrug resistant bacteria in the pool water could not be determined. The question of whether patients who are proven carriers of multidrug resistant bacteria may use therapy pools has to be clarified in individual cases, taking the respective bacterial species into account. In the case of unsafe operation or outdated technology and patients colonized with 4MRGN, a ban on use should be considered.

## 5. Conclusions

Despite the reduction of antibiotic-resistant bacteria due to water treatment and disinfection, some antibiotic-resistant bacteria are still present in the water of therapy pools and on surrounding surfaces. There they can potentially persist and infect other patients and staff alike. Adequate pool water treatment and management of cleaning and cleaning equipment can prevent the transmission of these bacteria. The capacity of the water treatment defines the maximum bathers load. This maximum number of visitors should not be exceeded to ensure good water quality.

## Figures and Tables

**Table 1:** Sampling sites of water samples (left column) and surrounding surfaces (right column).

Water Samples	No.	Volume	Sampling Points in the Pool Surroundings	No.
pool water	1	1000 mL	swimming aid	1
filtrate	2	100 mL	seats	2
balance tank water	3	100 mL	spillway	3
filter backwash water	4	100 mL	hand rail	4
			toilet	5
			shower rooms	6
			barefoot areas	7
			cleaning trolley	8
			cleaning equipment	9

**Table 2:** Parameter limits are according to DIN 19643-1 [21]. Limits are only valid for pool water and filtrate (not for balance tank water and filter backwash water).

Microbial Parameter	Method	Parameter Limits	Chemical Parameter	Method	Parameter Limits
Total heterotrophic counts at 36 °C	TrinkwV, appendix 5	<100 CFU/mL	THM (mg/L)	DIN EN ISO 10301:1997	<0.02
*Escherichia coli*	DIN EN ISO 9308-2	<1 CFU/100 mL	Bromate (mg/L)	DIN EN ISO 15061	<2.0
*Pseudomonas aeruginosa*	DIN EN ISO 16266	<1 CFU/100 mL	Chlorate and chlorite (mg/L)	LC-MS	<30
			Al (mg/L)	DIN EN ISO 11885	<0.05
			Fe (mg/L)	DIN EN ISO 11885	<0.02
			pH	DIN EN ISO 10523	6.5–7.5
			K_S4.3_ (mmol/L)	DIN 38409-1	>0.7
			Nitrate (mg/L)	EN ISO 10304:1995	<20
			Ox. (mg/L)	DIN EN ISO 8467	<0.75
			Redox (mV)	DIN 38404-6	>750
			Free chlorine (mg/L)	DIN EN ISO 7393-1	0.3–0.6
			Bound chlorine (mg/L)	DIN EN ISO 7393-1	<0.2

THM: Trihalogenmethane; LC-MS: Liquid chromatography–mass spectrometry.

**Table 3:** Chemistry in pool water samples (according to DIN 19643-1) and number of visitors per year (date from the questionnaire).

Hospital Number	1	2	3	4	5	6	7	8	9	10	11
Visitors/per year	2500	7200	4500	4500	10,800	1680	1450	35,000	3000	<50	300
THM (mg/L)	0.0	0.007	0.006	0.000	0.005	0.000	0.001	0.008	0.000	0.002	0.000
Bromate (mg/L)	0.055	<LOD	<LOQ	0.009	0.011	0.054	0.003	0.005	0.005	0.004	0.005
Chlorate and chlorite (mg/L)	18.0	2.0	0.8	3.2	2.1	33.5 ^a^	9.0	4.7	5.4	16.1	9.2
Al (mg/L)	0.06 ^a^	0.07 ^a^	<LOQ	0.07 ^a^	<LOQ	0.07 ^a^	<LOQ	0.08 ^a^	<LOQ	0.1 ^a^	0.08 ^a^
Fe (mg/L)	<LOQ	<LOQ	<LOQ	<LOQ	<LOQ	<LOQ	<LOQ	<LOQ	<LOQ	0.0	<LOQ
pH	6.7	7.0	7.0	7.2	7.3	6.7	7.1	7.2	6.7	7.2	7.5
K_S4.3_ (mmol/L)	0.3 ^b^	0.9	0.6 ^b^	0.9	0.71	0.2 ^b^	0.4 ^b^	1.0	0.2 ^b^	0.5 ^b^	1.3
Nitrate (mg/L)	10.8	7.5	28.8 ^a^	26.8 ^a^	14.3	30.0 ^a^	<NG	3.5	13.3	30.7 ^a^	14.3
Ox (mg/L)	0.7	0.5	0.7	0.5	1.4	<LOD	0.4	0.9 ^a^	0.3	0.3	1.0 ^a^
Redox (mV)	780.80	821.50	806.00	738.30 ^b^	819.30	767.00	675.00 ^b^	716.30 ^b^	823.00	803.30	919.00
Free chlorine (mg/L)	0.51	0.72 ^a^	0.40	0.27 ^b^	0.50	0.33	0.54	0.77 ^a^	0.46	0.49	0.59
Bound chlorine (mg/L)	0.11	0.15	0.06	0.06	0.06	0.01	0.03	0.25 ^a^	0.04	0.02	0.02

<LOD = below limit of detection; <LOQ = below limit of quantification; ^a^ above requirements acc. DIN 19643-1; ^b^ below requirements acc. DIN 19643-1 [21].

**Table 4:** Number of positive samples from contact agar plates and percentage of positive samples (*n* = 370).

Hospital Number	1	2	3	4	5	6	7	8	9	10	11	% Positive Samples
swimming aid	4	2	2	4	4	1	2	1	3	3	3	66
seats	4	4	0	4	4	2	4	2	4	3	3	77
spillway	1	3	4	1	4	1	1	1	1	2	1	45
hand rail	4	4	2	4	4	2	4	2	4	2	3	80
toilet	3	3	1	4	3	2	3	2	4	3	3	70
shower rooms	2	2	1	4	4	2	4	2	3	3	3	68
barefoot areas	4	3	3	4	3	2	4	1	4	3	3	77
cleaning trolley	3	3	4	3	2	1	3	1	3	2	ND	57
cleaning equipment	1	4	3	4	3	2	4	2	4	4	ND	70
% positive samples	72	78	56	89	86	83	81	78	83	69	70	

ND = not determined.

**Table 5:** Antibiotic resistance patterns of all *Pseudomonas aeruginosa*, *Stenotrophomonas maltophilia*, and *Sphingomonas paucimobilis* isolates from water and surface samples.

Class of Antibiotics	Antibiotic															
Penicillins	ampicillin	r	r	r	r	r	r	r	s	r	r	r	r	r	s	s
	piperacillin	s	s	s	s	r	r	r	s	s	r	s	s	r	r	s
	piperacillin-tazobactam	s	s	s	s	r	r	r	s	s	s	s	s	r	r	s
Penicillins/Beta-lactamase inhibitor	amoxicillin-clavulanic acid	r	r	r	r	r	r	r	r	r	r	r	r	r	r	r
Cephalosporins (2nd)	cefuroxime	r	r	r	r	r	r	r	r	r	r	r	r	r	r	r
Cephalosporins (3rd)	cefotaxime	r	r	r	r	r	r	r	r	r	r	r	r	r	r	r
Cephalosporins (3rd)	ceftazidime	s	s	s	s	r	r	r	s	s	s	s	s	r	s	s
Cephalosporins (4th)	cefepime	s	s	s	s	r	r	r	s	s	s	s	s	r	s	s
Carbapenems	imipenem	s	r	s	r	r	r	r	r	r	r	r	r	r	s	s
	ertapenem	r	r	r	r	r	r	r	r	r	r	r	r	r	r	r
Aminoglycosides	gentamicin	s	s	s	r	r	r	r	r	r	r	r	r	s	s	s
	tobramycin	s	s	s	s	r	r	r	r	r	r	r	r	s	s	r
	amikacin	s	s	s	s	r	r	r	r	r	r	r	r	s	s	s
Fluoroquinolones	ciprofloxacin	s	r	r	s	s	r	r	s	s	s	s	r	r	s	s
	levofloxacin	s	r	r	s	s	s	r	s	s	s	s	s	r	s	s
Sulfonamide antibiotic	trimethoprim-sulfam.	r	r	r	r	s	s	s	r	r	r	s	r	s	s	s
Fosfomycin	fosfomycin	ND	ND	ND	ND	r	r	r	s	s	r	s	r	s	r	r
Glycylcycline	tigecycline	r	r	r	r	r	r	r	ND	ND	ND	ND	ND	ND	ND	ND
**Number of Isolates**		16	3	2	2	2	13	24	6	2	1	1	1	1	1	1
**Species**		*Pseudomonas aeruginosa* (26)				*Stenotrophomonas maltophilia* (39)			*Sphingomonas paucimobilis* (14)							

Susceptibility (s) and resistance (r) were automatically determined with the BD Phoenix™ System; ND = Not determined.

**Table 6:** Antibiotic resistance patterns of all *Achromobacter* spp. and *Sphingobacterium* spp. isolates from water and surface samples.

Class of Antibiotics	Antibiotic													
Penicillins	ampicillin	r	r	ND	r	r	r	r	r	r	r	r	r	s
	piperacillin	s	s	s	s	s	s	r	s	s	r	r	r	s
	piperacillin-tazobactam	s	s	s	s	s	r	s	s	s	s	r	s	s
Penicillins/Beta-lactamase inhibitor	amoxicillin-clavulanic acid	r	r	r	r	r	r	r	r	r	r	r	r	r
Cephalosporins (2nd)	cefuroxime	r	r	r	r	r	r	r	r	r	r	r	r	r
Cephalosporins (3rd)	cefotaxime	r	r	r	r	r	r	r	r	r	r	r	r	r
Cephalosporins (3rd)	ceftazidime	s	s	s	s	r	r	r	s	s	r	r	r	s
Cephalosporins (4th)	cefepime	s	s	s	r	r	r	r	s	s	s	r	s	s
Carbapenems	imipenem	ND	ND	ND	ND	ND	ND	ND	r	s	s	s	r	s
	ertapenem	ND	ND	ND	ND	ND	ND	ND	r	r	r	r	r	r
Aminoglycosides	gentamicin	s	s	r	r	r	r	r	s	r	r	r	r	s
	tobramycin	s	s	r	r	r	r	r	r	r	r	r	r	r
	amikacin	s	s	s	r	r	r	r	s	r	r	r	r	s
Fluoroquinolones	ciprofloxacin	s	r	r	r	r	r	r	s	s	s	s	s	s
	levofloxacin	s	r	s	r	r	r	s	s	s	s	s	s	s
Sulfonamide antibiotic	trimethoprim-sulfam.	s	s	s	s	s	s	s	s	s	s	s	s	s
Fosfomycin	fosfomycin	r	r	r	r	r	r	r	r	r	r	r	r	r
Glycylcycline	tigecycline	ND	ND	ND	r	r	r	ND	ND	ND	r	ND	r	ND
**Number of Isolates**		1	2	1	10	3	1	1	1	4	1	3	2	1
**Species**		*Achromobacter* spp. (19)							*Sphingobacterium* spp. (11)					

Susceptibility (s) and resistance (r) was automatically determined with the BD Phoenix™ System; ND = Not determined.

**Table 7:** MIC50 and MIC90 values of *Pseudomonas aeruginosa*, *Stenotrophomonas maltophilia*, and *Sphingomonas paucimobilis* isolates from water and surface areas.

Class of Antibiotics	Antibiotic	*Pseudomonas aeruginosa*		*Stenotrophomonas maltophilia*		*Sphingomonas paucimobilis*	
		MIC50	MIC90	MIC50	MIC90	MIC50	MIC90
Penicillins	ampicillin	16	16	16	16	8	16
	piperacillin	4	8	32	32	4	32
	piperacillin-tazobactam	4/4	8/4	32/4	32/4	4/4	29.2/4
Penicillins/beta-lactamase inhibitor	amoxicillin-clavulanic acid	64/2	64/2	64/2	64/2	32/2	60.8/2
Cephalosporins (2nd)	cefuroxime	16	16	16	16	16	16
Cephalosporins (3rd)	cefotaxime	8	8	8	8	8	8
Cephalosporins (3rd)	ceftazidime	2	4	8	16	4	8
Cephalosporins (4th)	cefepime	4	4	16	16	1	2
Carbapenems	imipenem	2	8	16	16	16	16
	ertapenem	2	2	2	2	2	2
Aminoglycosides	gentamicin	1	2	8	8	8	8
	tobramycin	1	1	8	8	8	8
	amikacin	4	4	32	32	32	32
Fluoroquinolones	ciprofloxacin	0.25	2	2	2	0.5	0.9
	levofloxacin	1	4	1	4	0.5	0.5
Sulfonamide antibiotic	trimethroprim-sulfam.	4/76	8/76	1/19	1/19	4/76	7.6/76
Fosfomycin	fosfomycin	ND	ND	>64	>64	32	64
Glycylcycline	tigecycline	4	4	1	2	ND	ND

The MIC values used for calculation of the MIC50 and MIC90 were determined with the BD Phoenix^TM^ System; ND = not determined.

**Table 8:** MIC50 and MIC90 values of *Achromobacter* spp. and *Sphingobacterium* spp. isolates from water and surface areas.

Class of Antibiotics	Antibiotic	*Achromobacter* spp.		*Sphingobacterium* spp.	
		MIC50	MIC90	MIC50	MIC90
Penicillins	ampicillin	16	16	16	16
	piperacillin	4	5.2	16	32
	piperacillin-tazobactam	4/4	4/4	8/4	32/4
Penicillins/Beta-lactamase inhibitor	amoxicillin-clavulanic acid	16/2	64/2	4/2	4.4/2
Cephalosporins (2nd)	cefuroxime	16	16	16	16
Cephalosporins (3rd)	cefotaxime	8	8	8	8
Cephalosporins (3rd)	ceftazidime	8	16	4	16
Cephalosporins (4th)	cefepime	16	16	1	16
Carbapenems	imipenem	ND	ND	4	8
	ertapenem	ND	ND	2	2
Aminoglycosides	gentamicin	8	8	8	8
	tobramycin	8	8	8	8
	amikacin	32	32	32	32
Fluoroquinolones	ciprofloxacin	2	2	0.25	0.5
	levofloxacin	2	4	0.5	0.5
Sulfonamide antibiotic	trimethoprim-sulfam.	1/19	1/19	1/19	1/19
Fosfomycin	fosfomycin	>64	>64	>64	>64
Glycylcycline	tigecycline	2	2	1 *	1 *

The MIC values used for calculation of the MIC50 and MIC90 were determined with the BD Phoenix^TM^ System; ND = not determined. * calculated from three values.

## Supplementary Materials

The following are available online at http://www.mdpi.com/1660-4601/15/12/2666/s1, S1: questionnaire for the study “resistant pathogens in hospital swimming pools”, S2: raw data.
