# Supplementary material for: Occurrence of Antibiotic-Resistant Bacteria in Therapy Pools and Surrounding Surfaces
Source: Int J Environ Res Public Health. 2018 Nov 27;15(12):2666. doi: 10.3390/ijerph15122666 (PMC6313675; doi:10.3390/ijerph15122666)
Supplement: Supplementary file 1 [file ijerph-15-02666-s001.zip › S1 Questionnaire.pdf]

Questionnaire for the study "Resistant pathogens in hospital swimming pools"

1. Information about the hospital:

Hospital name:	
Hospital plan number:	
Street:	
Postal code:	
Number of beds:	

Contact person:	Pool:	Hospital hygienist:	Hygiene specialist
Titel:			
Surname:			
Name:			
Telefone:			
Fax:			
E-Mail:			

2. Information on the use of the bath:

Ø Visitor per day:
Ø Visitor per year:
Estimated person frequency (persons per h):
Nominal load of the bath (1 / h):

Which patient groups use the bath?
- Surgical yes ☐ no ☐
- Internal medicine yes ☐ no ☐
- Others yes ☐ no ☐

Are certain patients excluded from use? yes ☐ no ☐

If yes, specify:
- Gastroenteritis yes ☐ no ☐
- Open wounds yes ☐ no ☐
- MRE-Carrier yes ☐ no ☐
- Others

Extern unsers: yes ☐ no ☐

Baby swimming: yes ☐ no ☐

other events:

3. **Type and number of pools (one column per pool for several pools):**

Type of pool (DIN 19543-1 Point 7)				
Construction year				
Last modernisation (year)				
Pool size (m^2)				
Pool volume (m^3)				
Flow (m^3/h)				
circulation time (min)				
Temperature (°C)				
Ø pH value				

---

4. **Water treatment and origin:**

Process combination with fixed bed and precoat filters

Flocculation - Filtration - Chlorination ☐

Adsorption on activated carbon - Flocculation - Filtration - Chlorination ☐

Flockung - Multi-layer filtration with adsorptive carbon - Chlorination ☐

Flocculation - Filtration - Adsorption on grain activated carbon - Chlorination ☐

Flocculation - Filtration - UV-Desinfection - Chlorination ☐

Adsorption on activated carbon --- precoat filtration --- Chlorination ☐

Process combination with ozonation

Flocculation - Filtration –Ozonation- Chlorination ☐

Flocculation - Ozonation - Multi-layer filtration – Chlorination ☐

Process combination with ultrafiltration ☐

filter flushing automatically ☐ manually ☐

Frequency of filter flushing: x per ☐ Day ☐ Week ☐Month

Origin filling water

Public ☐

Own source ☐

Filling water (m^3/d):

Desinfection

Disinfectant:

Documentation yes ☐ no ☐

Microbiological controls according DIN 19643
1x per month ☐
More often ☐
Less often ☐

Chemical controls according DIN 19643
1x per month ☐
More often ☐
Less often ☐

Handling of fecal contamination or abnormal microbiological findings (e.g., high chlorination)):

---

5. **Technical details:**

Automatic measuring and control technology yes ☐ no ☐

Last maintenance / calibration:
Last filter change:

6. General hygiene

Ventilation system available yes ☐ no ☐

Cleaning and disinfection plan available yes ☐ no ☐

As part of the hygiene plan yes ☐ no ☐

Which cleaning devices are used?

Scrubbers and cleaning brushes yes ☐ no ☐
Mop / flat mop yes ☐ no ☐
Brush roller machine yes ☐ no ☐
high pressure cleaner yes ☐ no ☐
Wet vacuum / dry vacuum cleaners yes ☐ no ☐

Proper cleaning and disinfection of the devices after use? yes ☐ no ☐

Who carries out the purification of the cleaning utensils?

Subsequent sufficient drying guaranteed? yes ☐ no ☐

Frequency of change for cleaning utensils set in the hygiene plan? yes ☐ no ☐
Storage of cleaning utensils and equipment
dry yes ☐ no ☐
wet yes ☐ no ☐

Regular cleaning checks

Visuall yes ☐ no ☐

Chemical (e.g. Glowcheck) yes ☐ no ☐

Mikrobiological yes ☐ no ☐

sanitary facilities

Number of showers Women:
Men:
Number of toilets Women:
Men:

Carrying out of cleaning and disinfection measures

	Cleaning substance and concentration	cleaning interval (e.g., occasion, daily, weekly, etc.)	Disinfectant substance and concentration	desinfection interval (e.g., occasion, daily, weekly, etc.)
Pool floor				
Pool walls				
Spillway				
Balance water tank				
Water-tank				
Seats / Furniture				
Barefoot areas				
Shower				
Toilet				

7. Personnel

Qualified specialists yes ☐ no ☐

Master for baths yes ☐ no ☐
Specialist for baths yes ☐ no ☐

At least annual instruction of the cleaning staff in the cleaning and disinfection plan
yes ☐ no ☐

Use of external cleaning personnel yes ☐ no ☐

8. Swimming aids and physiotherapy equipment

Use of swimming aids and physiotherapy equipment (e.g., noodles) yes ☐ no ☐

Regular cleaning of swimming aids and physiotherapy equipment yes ☐ no ☐

Regular desinfection of swimming aids and physiotherapy equipment yes ☐ no ☐

Storage of swimming aids and physiotherapy equipment
dry yes ☐ no ☐
wet yes ☐ no ☐

Comments:

Date: Signature:
